# Pediatric Complicated Pneumonia Caused by *Streptococcus pneumoniae* Serotype 3 in 13-Valent Pneumococcal Conjugate Vaccinees, Portugal, 2010–2015

**DOI:** 10.3201/eid2407.180029

**Published:** 2018-07

**Authors:** Catarina Silva-Costa, Maria João Brito, Marcos D. Pinho, Ana Friães, Sandra I. Aguiar, M. Ramirez, Jose Melo-Cristino, on behalf of the Portuguese Group for the Study of Streptococcal Infections, the Portuguese Study Group of Invasive Pneumococcal Disease of the Pediatric Infectious Disease Society

**Affiliations:** Instituto de Microbiologia, Instituto de Medicina Molecular, Faculdade de Medicina, Universidade de Lisbon, Lisbon, Portugal (C. Silva-Costa, M.D. Pinho, A. Friães, S.I. Aguiar, M. Ramirez, J. Melo-Cristino);; Centro Hospitalar de Lisboa Central, Lisbon (M.J. Brito)

**Keywords:** Streptococcus pneumoniae, conjugate vaccines, serotype, empyema, pneumonia, Portugal, 13-valent pneumococcal conjugate vaccine, PCV13, serotype 3, vaccination, immunization, vaccine-preventable diseases, bacteria, bacterial infection, respiratory infections, vaccine failure

## Abstract

Despite use of 7-valent pneumococcal conjugate vaccine, incidence of pleural effusion and empyema (pediatric complicated pneumococcal pneumonia [PCPP]) is reportedly increasing globally. We cultured and performed PCR on 152 pleural fluid samples recovered from pediatric patients in Portugal during 2010–2015 to identify and serotype *Streptococcus pneumoniae*. We identified only 17 cases by culture, but molecular methods identified *S. pneumoniae* in 68% (92/135) of culture-negative samples. The most frequent serotypes were 3, 1, and 19A, together accounting for 62% (68/109) of cases. Nineteen cases attributable to 13-valent pneumococcal conjugate vaccine (PCV13) serotypes (mostly serotype 3) were detected among 22 children age-appropriately vaccinated with PCV13. The dominance of the additional serotypes included in PCV13 among PCPP cases in Portugal continues, even with PCV13 available on the private market (without reimbursement) since 2010 and with average annual coverage of 61% among age-eligible children. Our data suggest reduced effectiveness of PCV13 against serotype 3 PCPP.

*Streptococcus pneumoniae* (pneumococcus) is the leading cause of bacterial pneumonia in children and is the most common pathogen isolated in pleural effusions and empyemas (*1*–*3*). In this article, we will refer to pediatric pneumococcal pneumonias occurring with either parapneumonic effusion or empyema as pediatric complicated pneumococcal pneumonias (PCPPs). Several studies reported an increasing incidence of PCPP in the 1990s and early 2000s (*1*,*2*,*4*), a trend that was observed to persist or even accelerate after the introduction of the 7-valent pneumococcal conjugate vaccine (PCV7), which covered serotypes 4, 6B, 9V, 14, 18C, 19F, and 23F (*1*,*2*,*4*,*5*). Several factors could be responsible for an apparent increase in PCPP, including greater awareness and improved diagnostics (*1*). However, temporal trends or vaccine-induced changes in serotype prevalence and the particular propensity of certain serotypes to cause PCPP have also been implicated (*1*). Serotypes 1, 3, 7F, 14, and 19A are the dominant serotypes in PCPP (*1*), and some studies associated the increase in PCPP after the introduction of PCV7 with increasing incidence of PCPP by serotypes 1, 3, and 19A, which are not targeted by PCV7 (*1*,*2*,*6*).

Culture of pleural fluid or blood of PCPP case-patients is frequently negative; the yield of cultures is as low as 8% for pleural fluid and only slightly higher for blood (*7*,*8*). Although one of the reasons behind this low yield might be previous antimicrobial drug treatment (*9*,*10*), a recent study showed that serotype 3 is infrequently cultured from children’s samples but is readily detected by PCR-based assays, even without prior exposure to antibiotics (*10*). Although PCR-based assays for the direct detection of pneumococci from clinical samples have limitations (*11*), they also offer several advantages, such as speed, high sensitivity, and being independent of bacterial viability (*9*,*10*). Given these advantages, PCR-based techniques are increasingly being used as important tools in the diagnosis of pneumococcal invasive infections and the epidemiologic surveillance of the characteristics of unculturable pneumococci (*10*).

PCV7 was introduced in Portugal in 2001. PCV10, which added serotypes 1, 5, and 7F to the PCV7 serotypes, became available in mid-2009. PCV13, which added serotypes 3, 6A, and 19A to the PCV10 serotypes , became available in early 2010. All PCVs were offered on the private market without any reimbursement. Even without reimbursement, 75% of children born in 2008 were vaccinated with PCV7, although soon afterward a decline in vaccination coverage occurred; coverage reached 63% in 2012 (*12*) and remained stable at 61% until 2014 (unpublished data from IQVIA, Pfizer, and Instituto Nacional de Estatística, assuming a 4-dose schedule). In July 2015, PCV13 was introduced in the national immunization plan (NIP) for children born after January 2015. We conducted a prospective study to determine the serotypes causing PCPP in children in Portugal during 2010–2015, a period when PCV13 was being administered outside of the NIP.

## Materials and Methods

### Patient Samples

We asked 61 hospital laboratories and pediatric departments located throughout Portugal to report all cases of possible PCPP (in patients <18 years of age) for which pleural fluid was available for analysis and to submit these samples for characterization. We included in our study only samples recovered during January 2010–December 2015, but we performed no audit that would ensure reporting compliance. Because our network includes all secondary and tertiary care hospitals in which PCPP is likely to be treated and a pleural fluid sample obtained, we assume our catchment population is the entire population of Portugal <18 years of age. During 2010–2015, this population steadily decreased, from 1,929,331 in 2010 to 1,802,196 in 2015 (average 1,865,288) (https://www.ine.pt).

Whenever possible, the vaccination status of the patient was also reported. We used guidelines from the Vaccines Committee of the Pediatric Infectious Diseases Society and Portuguese Pediatric Society from 2014 (*13*) and 2009 (*14*) to define age-appropriately vaccinated children with PCV7 and PCV13 (Table 1). The study was approved by the Institutional Review Board of the Centro Académico de Medicina de Lisboa.

**Table 1:** PCV immunization schemes according to guidelines from the Vaccines Committee of the Pediatric Infectious Diseases Society and Portuguese Pediatric Society*

Vaccine	Start of vaccination scheme	Primary immunization course	Booster vaccination
PCV7	2–6 mo	3 doses, at 2, 4, and 6 mo	12–15 mo
7–11 mo	2 doses	Booster dose at 12 mo†
12–23 mo	2 doses‡	NA
24–59 mo	Risk group only: 2 doses	NA
PCV13	2–6 mo	2 or 3 doses§	11–15 mo¶
	7–11 mo	2 doses§	11–15 mo¶
	12–23 mo	2 doses‡	NA
	>24 mo	1 dose	NA

*NA, not applicable; PCV, pneumococcal conjugate vaccine; PCV7, 7-valent PCV; PCV13, 13-valent PCV. †8 weeks after the second dose. ‡>8 weeks apart. §Minimum interval between doses 4 weeks. ¶Minimum interval from last dose 8 weeks.

### Culture-Positive Samples

We identified all bacteria as *S. pneumoniae* by colony morphology, hemolysis on blood agar plates, optochin susceptibility, and bile solubility. We performed serotyping with the standard capsular reaction test by using the chessboard system and specific serum samples (Statens Serum Institute, Copenhagen, Denmark). For this study, we classified serotypes as vaccine serotypes (i.e., those in PCV7; the additional 3 serotypes included in PCV10 [addPCV10]; the additional 3 serotypes included in PCV13 [addPCV13]) and nonvaccine serotypes.

### Culture-Negative Samples

When identification of disease etiology by conventional microbiologic methods failed, we sent pleural fluid to the central laboratory, where total DNA was extracted from 200 μL of the patient sample by using the High Pure PCR Template Preparation Kit (Roche, Mannheim, Germany) or DNeasy Blood and Tissue Kit (QIAGEN, Hilden, Germany) according to the manufacturers’ instructions. We used a conventional PCR amplification of 2 human genes (encoding human β-actin and RNaseP) as control for the quality of the purified DNA. We initially tested the presence of *S. pneumoniae* by using conventional PCR to target the *lytA* and *wzg* genes (2010–2011) and later (2012 and later) by using real-time PCR (rPCR). We serotyped positive samples by conventional PCR, rPCR, or a combination of both. We confirmed negative results by conventional PCR by using rPCR on samples stored at −80°C.

### Conventional PCR

We used a multiplex PCR for the amplification of the genes encoding human β-actin and RNaseP (*15*,*16*) and *S. pneumoniae* genes (*lytA* and *wzg*) (*17*,*18*). We serotyped PCR-positive samples by using multiplex PCR, with primers targeting serotypes 1, 3, 4, 5, 6A, 6B, 6C, 7F, 8, 9V, 14, 15A, 15B/C, 16, 18C, 19A, and 23F (*17*,*19*). We performed PCR reactions in a final volume of 25 μL containing 4 μL of DNA, 1X polymerase buffer (Biotools, Madrid, Spain), 3U of polymerase GoTaq (Biotools, Madrid, Spain), 10 mmol/L dNTPs, and 10 pmol of each primer (with the exception of human β-actin primers, for which 7 pmol was used). The PCR program consisted of 4 min denaturation at 95°C, 30 cycles of 95°C for 40 s, 58°C for 40 s, and 65°C for 2 min, followed by a final extension at 65°C for 10 min.

### rPCR

We performed rPCR on the Rotor-Gene 6000 (Corbett Research, Cambridge, United Kingdom) by using the Platinum quantitative PCR SuperMix-UDG (Thermo Fisher Scientific, Waltham, Massachusetts, USA). PCR reactions contained 5 μL of DNA, 5 pmol/L of each primer (targeting *lytA* and *wzg* genes) (*18*,*20*), 2.5 pmol/L of each probe, 12.5 μL of PCR SuperMix-UDG, 1.5 μL of MgCl_2_ (50 nmol/L), and water, for a final volume of 25 μL. Cycling conditions were as follows: 95°C for 10 min, followed by 50 cycles at 95°C for 15 s and 60°C for 1 min. We performed detection of *lytA* and *wzg* in singleplex PCR. We defined negative results as those with cycle threshold (C_t_) >40 and positive results as those with C_t_ <35 (*21*). If a C_t_ value >35 and <40 was obtained, we considered the result inconclusive and varied the amount of DNA in the reaction by using twice as much DNA and diluting over a 50-fold range. If still no reaction yielded a C_t_ <35, we considered the sample negative.

For serotyping by rPCR (*22*), we performed 7 multiplex reactions targeting 3 serotypes or serogroups each: 3, 7F/7A, and 19A; 1, 15B/15C, and 23F; 14, 18C, and 19F; 4, 6, and 9V/9A; 5, 11A/11D, and 16F; 8, 12F/12A/12B, and 22F/22A; and 15A, 23A, and 33F/33A/37. We also used rPCR to serotype positive samples that tested negative for all serotypes by conventional PCR. The rPCR scheme for serotyping did not enable discrimination between some serotypes within a few serogroups, as indicated in the description of each multiplex reaction.

### Statistical Methods

We used the Fisher exact test to evaluate differences in the prevalence of the most frequent addPCV10 and addPCV13 serotypes, as well as the number of PCPPs among vaccinated and nonvaccinated children. We consider a p value <0.05 statistically significant. We report patient age as median years and interquartile range (IQR).

## Results 

### Patient Samples

During January 2010–December 2015, we analyzed 152 pleural fluid samples; 17 cases were identified by culture to have pneumococcal etiology by the participating laboratories. We submitted 135 culture-negative samples to the central laboratory for molecular testing. In 43 samples, we did not detect *S. pneumoniae* in the pleural fluid by molecular methods, but we confirmed the remaining 92 cases (68%) to be PCPP by molecular methods. The total numbers of requests for molecular testing and samples positive for *S. pneumoniae* were approximately constant over the years (Table 2).

**Table 2:** Number of requests for laboratory testing and confirmed positive cases of *Streptococcus pneumoniae* infection in children, Portugal, 2010–2015*

Cases and requests	Year						
	2010	2011	2012	2013	2014	2015	Total
Positive for *S. pneumoniae*	29	18	19	18	10	15	109
Negative for *S. pneumoniae*	2	3	8	10	6	14	43	
Requests	31	21	27	28	16	29	152

*Among the cases are 17 for which pneumococci were cultured from pleural fluid (2010, n = 5; 2011, n = 3; 2012, n = 2; 2013, n = 3; 2014, n = 0; and 2015, n = 4).

### PCPP Case-Patients

Among the 109 PCPP case-patients (17 identified by culture and 92 by molecular methods), 56 were male and 52 female; the sex of 1 patient was not available. Patient age ranged from 4 months to 17 years (median age 4 [IQR 2.3–6] years); 27 cases were in children <2 years of age. A total of 34 (31%) PCPP cases occurred in nonvaccinated children (median age 2.6 [IQR 1.4–8.5] years), and the vaccination status was unknown for 28 (26%) patients. The remaining 47 (43%) PCPP cases occurred in children who received >1 vaccine dose (median age 3.2 [IQR 3–5] years). All vaccinated children received PCV7, PCV13, or both; PCV10 had not been administered to any child in the study.

### Serotypes of PCPP Case-Patients

Except for 18 (16%) samples, we were able identify the serotypes of the pneumococci responsible for illness (Table 3). Overall, the most frequent serotype was serotype 3, responsible for 36% of the PCPP cases (n = 40; median patient age 3 [IQR 2–5] years), followed by serotype 1 (n = 21 [19% of cases]; median patient age 5.5 [IQR 4–13.5] years). Other serotypes found were 19A (n = 7); 7F/7A (n = 4); 14 (n = 3); 5, 16F, and 7F (n = 2 each); and 6B, 6, 8, 9V, 9V/A, 10A, 15A, 19F, 23F, and 33F/33A/37 (n = 1 each); median patient age for all of these cases was 3 (IQR 1–6) years. Although serotype 1 cases occurred more frequently among older children than serotype 3 cases (p = 0.005 by Fisher exact test), the age distribution of the case-patients infected with each of these serotypes was not significantly different from that of PCPP case-patients infected with all other serotypes (p>0.198 by Fisher exact test). Whenever possible, conventional PCR serotyping enabled the identification of serotypes 7F, 6B, and 9V, whereas rPCR detected only the groups 9V/9A, 7F/7A, and 6 without further discrimination. On the 17 culture-positive samples, only 5 serotypes were found: 1 and 3 (n = 7 each) and 14, 10A, and 19A (n = 1 each). Apart from serotype 10A, which was detected exclusively in 1 isolate, all other serotypes were also detected by PCR from culture-negative samples. Most of the PCPP case-patients for whom a serotype was unambiguously identified were infected with the addPCV13 serotypes (n = 47 [43%]), followed by the addPCV10 serotypes (n = 25 [23%]), whereas PCV7 serotypes were responsible for a small fraction of PCPP cases (n = 7 [6%]). Among the remaining cases, 6 were caused by nonvaccine serotypes, and in another 6 cases, we were unable to classify capsular types as vaccine or nonvaccine type. These cases included samples for which rPCR tested positive for serogroup 6 (n = 1), 7F/7A (n = 4), or 9V/9A (n = 1).

**Table 3:** Serotype distribution among the 152 pediatric case-patients with *Streptococcus pneumoniae* infection included in this study, by PCV vaccination status, Portugal, 2010–2015*

Serotype	Vaccination status						
	AP_PCV13	AP_PCV7	NA_PCV13	NA_PCV7	Not vaccinated	Unknown	Total
14	1				2		3
19F						1	1
23F						1	1
PCV7	1		1		2	2	6
1	1	3		2	7	8	21
5		1				1	2
PCV10	2	4	1	4	9	11	31
3	17	6		1	11	5	40
19A		1			5	1	7
PCV13	19	11	1	5	26	17	79
16F						2	2
8				1			1
15A					1		1
10A			1				1	
7F/7A		1		3†		2	6
6					2‡		2
9V/9A			1§		1		2	
33F/33A/37			1				1
Nonvaccine type		1	2	2	3	4	12
Not identified	3	1	1	1	5	7	18
Total	22	13	4	8	34	28	109


*AP_PCV13, age-appropriately vaccinated with PCV13; AP_PCV7, age-appropriately vaccinated with PCV7; NA_PCV13, not age-appropriately vaccinated with PCV13; NA_PCV7, not age-appropriately vaccinated with PCV7; PCV, pneumococcal conjugate vaccine; PCV7, 7-valent PCV; PCV13, 13-valent PCV. †Serotype 7F (n = 2). ‡Serotype 6B (n = 1). §Serotype 9V (n = 1).

### Vaccination Status of PCPP Case-Patients

Among the 47 vaccinated children, 35 were age-appropriately vaccinated (Table 3). Of these 35 children (median age 4 [IQR 5.5–6] years), 13 had received 4 doses of PCV13 and 13 (median age 5 [IQR 3–5] years) had received 4 doses of PCV7; the remaining children had received fewer doses (n = 6) or a combination of both PCVs (n = 3). Among the 22 children age-appropriately vaccinated with PCV13, 19 had infections caused by serotypes included in PCV13, representing vaccine failures. These cases occurred throughout the study period (1 case in 2010, 2 in 2012, 9 in 2013, and 7 in 2015). Among this group were 12 children (median age 3 [IQR 3–3.5] years) who had completed a 4-dose scheme of PCV13; 11 had serotype 3 PCPP and 1 serotype 1 PCPP. We also identified serotype 3 in 4 of 6 PCPPs in children who were age-appropriately vaccinated and had received 3 doses of PVC13 (1 case in a 1.5-year-old child, 2 in 2-year-old children, and 1 in a 3-year-old child). Among children who received a combination of both conjugate vaccines, a series of 3 or 4 doses of PCV7 followed by 1 dose of PCV13 serotype 3 was detected in 2 case-patients (both 6-year-olds) and serotype 14 in 1 case-patient (a 3.5-year-old). Among the 13 children age-appropriately vaccinated with PCV7 (median age 5 [IQR 3–5] years), all had received 4 PCV7 doses, and we detected no cases caused by PCV7 serotypes. Most cases were caused by addPCV13 serotypes; serotype 3 was most prevalent (n = 6), followed by serotype 1 (n = 3), and serotypes 5 and 19A (n = 1 each).

We compared the number of addPCV10 and addPCV13 PCPP cases (i.e., those caused by serotypes 1, 3, 19A, and 7F) among children age-appropriately vaccinated with PCV13 and among the other case-patients for whom vaccination status was known and serotype could be unambiguously determined. Serotype 3 was overrepresented among PCPP cases in children age-appropriately vaccinated with PCV13 (p<0.001 by Fisher exact test). We detected a similar number of positive samples for *S. pneumoniae* among children age-appropriately vaccinated with PCV13 (n = 22/27) and among the other case-patients for whom vaccination status was known (n = 59/71) (p = 1 by Fisher exact test).

## Discussion

The detection of *S. pneumoniae* in pleural fluid samples was greatly improved by the use of molecular methods, as previously reported (*3*); only 17 culture-positive cases of 109 were confirmed PCPPs (16%). Serotype 3 and the other major serotypes found (1, 19A, 7F/7A, and 14) have already been associated with complicated pneumonias and pneumococcal empyemas worldwide (*3*,*9*,*23*–*26*). Serotypes 1, 19A, and 14 were important causes of pediatric invasive pneumococcal disease (IPD) in Portugal during 2008–2012 (*12*) and were also among the most prevalent in PCPPs; however, serotype 3, which accounted for a small fraction of pediatric IPD cases during 2008–2012 (n = 9 [2%]) was the most frequent serotype in PCPPs. Although several factors might explain this difference, serotype 3 isolates might be more prone to specifically invade the pleural space and cause complicated pneumonia, with or without empyema. Supporting this hypothesis, a previous study found that pneumonia caused by serotype 3 was associated with an increased risk for necrotizing pneumonia, associated parapneumonic empyema, and increased severity of illness (*25*).

In the group of children vaccinated with PCV13, the most frequent serotype was 3, despite the potential protection conferred by vaccination. In fact, serotype 3 cases were more prevalent among children age-appropriately vaccinated with PCV13 than among the other case-patients for which vaccination status was known. One possible explanation for this could be a more limited protection of PCV13 against serotype 3 PCPP and a more effective protection against other serotypes, namely the other most prevalent serotypes (1, 7F and 19A), which would increase the likelihood that any PCPP cases in this group would be caused by serotype 3.

The effectiveness of PCV13 against serotype 3 has been questioned in several studies. In a large surveillance study performed in the United States, no reduction in IPD caused by serotype 3 was observed despite reductions in IPD incidence and evident decreases in IPD caused by PCV13 serotypes 19A and 7F (*27*). A postlicensure indirect cohort study in England, Wales, and Northern Ireland to assess vaccine effectiveness against IPD indicated that, for serotype 3, the calculated correlate of protection was 2.83 μg/mL, which is much higher than the 0.35 μg/mL aggregate correlate of protection used during licensing, suggesting a potential explanation for the reduced effectiveness of PCV13 against this serotype (*28*). The cases of serotype 3 PCPP in our study among children age-appropriately vaccinated with PCV13 occurred mostly in children >3 years of age (n = 12/17), but the distribution was similar to that of serotype 3 case-patients not vaccinated with PCV13 (p = 0.353 by Fisher exact test), so it does not seem likely that this was attributable to faster waning of the immune response to this serotype. In fact, cases of serotype 3 PCPP occurred in younger children than did cases caused by serotype 1, a serotype also included in PCV13 and for which only 1 vaccine failure was detected. Because the synthesis of the serotype 3 capsular polysaccharide proceeds through a synthase mechanism, the polysaccharide is not covalently linked to the peptidoglycan and can be released during growth, thereby potentially reducing opsonophagocytosis (*29*,*30*). Free capsular polysaccharide in pleural effusions in vitro can also neutralize type-specific anticapsular antibody, further reducing the efficacy of antibody-mediated clearance (*26*), and the considerable amounts of capsule produced could further enhance these effects in serotype 3 strains. Taken together, these data suggest that PCV13 might offer more limited individual protection against serotype 3, particularly in the context of complicated pneumonia.

The proportion of children asymptomatically colonized with serotype 3 increased in the period preceding PCV13 introduction in Portugal (*31*), but no data are available for more recent years. However, serotype 3 is currently the most important serotype among pneumococcal infections in adults (*32*,*33*), suggesting substantial circulation of these strains. Even so, if PCV13 in the NIP reduces colonization by serotype 3 isolates, its overall effectiveness could be much higher than the individual protection afforded because a reduction of the circulation of serotype 3 would also mean less opportunities to cause infection. Such an effect could be behind the decrease in PCPP observed with the introduction of PCV13 in several countries (*2*,*5*).

In agreement with our observations, other reports document serotype 3 vaccine failures. A study in Greece found 5 cases of complicated pneumonia caused by serotype 3 pneumococci among vaccinated children, although most vaccine failures occurred in children who received a single dose of PCV13, which could offer only limited protection (*34*). A more recent report, also from Greece, found 4 cases of empyema caused by serotype 3 pneumococci among children vaccinated with either a 3-plus-1 schedule (n = 3) or a booster dose at the age of 21 months (n = 1) (*35*), which can be considered vaccine failures. A study from Catalonia, Spain, also found 9 vaccine failures among 86 cases of IPD, mostly caused by serotype 3 (n = 6), including 2 cases in children who had received 4 vaccine doses (*36*).

Although our study was prospective and involved both pediatric and microbiology departments, it was not designed to estimate the incidence of PCPP because it did not identify cases in which there were clinical or radiographic criteria for complicated pneumonia and for which pneumococci were identified in either blood or respiratory samples. Although this certainly resulted in an underestimation of PCPP, the use of conventional PCR and rPCR techniques greatly enhanced the ascertainment of PCPP cases by improving the detection of *S. pneumoniae* in pleural fluid samples and emphasizes the potential role of molecular techniques when evaluating disease incidence. Another limitation of our study is lack of detailed information about the immune status or other underlying conditions in age-appropriately vaccinated children with PCPP by vaccine serotype. However, given the high prevalence of serotype 3 in this group, it is unlikely that all cases could be explained by host characteristics, indicating that specific properties of serotype 3 must be responsible for this behavior.

In summary, we describe data that are compatible with a lower individual effectiveness of PCV13 against PCPP, a presentation of IPD for which PCV13 is specifically recommended. The public health consequences of such possible lower protection might be mitigated by a reduction in circulating serotype 3 by vaccination with very high coverage, such as those achieved through inclusion in NIPs. Carriage studies and continued surveillance are necessary to determine the group effect of the introduction of PCV13 in the NIP to clarify the effectiveness of PCV13 in the prevention of infections caused by serotype 3.
